# Desloratadine Induced Pill Esophagitis

**DOI:** 10.4021/gr398w

**Published:** 2012-01-20

**Authors:** Huseyin Alkim, Mustafa Iscan

**Affiliations:** a Gastroenterology Specialist, Alman Hospital, Gastroenterology Department, Istanbul, Turkey

**Keywords:** Antihistamines, Desloratadine, Endoscopy, Pill esophagitis

## Abstract

Pill induced esophagitis is a rare complication mostly seen in patients using tetracycline and its derivatives or non-steroidal anti-inflammatory drugs. Here we present a 37 years old female patient experiencing pill esophagitis after taking desloratadine without liquid immediately before going to bed. This was the first pill esophagitis case related with desloratadine reported in the literature. Pill esophagitis is a preventable complication that consists of giving simple advice of how and when to take medication.

## Introduction

Pill-induced or drug-induced esophagitis is a rare clinical disorder. More than one hundred drugs have been reported to have the potential to induce esophageal lesions i.e. pill esophagitis. Pill esophagitis seems to be more common than expected especially in young women taking doxycycline capsules, but it is also associated with non-steroidal anti-inflammatory drugs, slow-release forms of potassium chloride, ferrous sulphate, and some other drugs. The symptoms of pill esophagitis are variable but include acute onset of pain on swallowing (odynophagia), persistent retrosternal pain and dysphagia. Endoscopic features include erosions, deeper esophageal ulcers and focal or more widespread inflammation. Common places to find ulcerations from pill esophagitis are where there is anatomic narrowing, including the level of the arch of the aorta, the left main stem bronchus, and the gastro-esophageal junction [1-4]. Here we present a case of pill esophagitis related with desloratadine.

## Case Report

**Figure 1:**
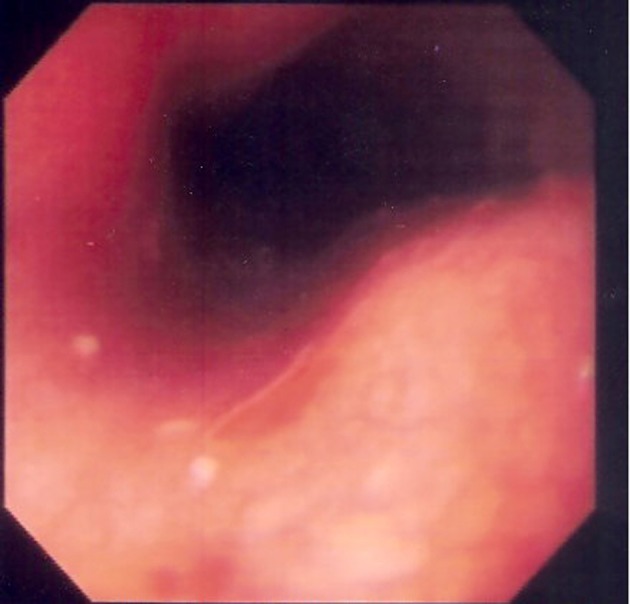
Endoscopic photograph showing erosion at the middle esophagus.

A 37 years old female patient was admitted to our clinic with the complaints of odynophagia and dysphagia together with retrosternal pain radiating to her back for last 5 days. Her past medical history was unremarkable for upper gastrointestinal disease. At upper gastrointestinal endoscopic examination, a 5 - 6 mm erosion was seen at the mid-esophagus, 32 cm from the front cutting teeth, the level where the main stem bronchus narrow the esophagus (Fig. 1). The rest of the esophageal mucosa, Z-line, cardia, stomach and duodenum appeared normal endoscopically. When her history was deepened, we learned that, the night before her complaints she had take one tablet of desloratadine (aerius® 5 mg film tablet) with little water just before going to bed. And in the morning she was awake with complaints. She was prescribed proton pump inhibitor together with sucralfate and dietary recommendations (liquid-soft diet, avoiding hot, cold and acidic drinks or foods). Her complaints were resolved in 10 days.

## Discussion

In any case with proximal esophagitis pill induced esophagitis must be suspected. The site most frequently affected was presumed to be in the mid-third of the esophagus, as in our case. However, pill esophagitis can be located at any level where the esophagus is narrowed: the upper sphincter, the aortic arch, enlarged left atrium, the left main bronchus and the lower esophageal sphincter. The size of the pill appears to influence the localization of the lesion; that is proximal in large-sized pills and distal in small-sized ones. In its distal location, pill esophagitis is often mistaken with reflux esophagitis [1-4].

Since first description of pill esophagitis in 1970, more than 1000 cases have been described and more than 100 drugs have been reported to have the potential to induce esophagitis. More than 90 % of reported cases were related with the use of antibiotics (especially tetracycline and its derivatives), non-steroidal anti-inflammatory drugs, potassium chloride, quinidine and biphosphonates. There are only two reports in the literature which describes pill esophagitis due to administration of antihistamines. The first one is an old report describing thiazinamium induced esophageal ulcerations [5]. The second report was related with claritine-D 24 hour extended release tablets which contains both loratadine and pseudoephedrine [6]. To our knowledge, this is the first desloratadine related pill esophagitis reported in the literature.

Many risk factors for pill esophagitis have been discussed, such as patient, esophageal and drug factors. Data indicate that anyone that takes medication is at risk of esophageal injury [7]. Taking medication with inadequate water or in supine position and lying down immediately after ingestion of the drug were the most important patient factors. Esophageal factors are the dysmotility and physiologic or pathologic narrowings' of the esophagus. Lastly, intrinsic caustic characteristics, solubility, adhesiveness, sustained-release formulations and contact time are the drug related factors which influence the type and severity of the esophageal injury.

Endoscopy is the method of choice for the diagnosis of pill esophagitis. A large spectrum of esophageal abnormalities has been reported, ranging from reddened edematous mucosa, with small superficial ulcerations, to a large ulcer with inflamed margins and profuse exudate. Stricture, mass and bleeding have also been reported [8].

Patients with pill induced esophageal injury typically take their medication with little or no water immediately before going to bed. They may then wake up two or three hours later with severe retrosternal chest pain and odynophagia. This could be prevented by swallowing the pills with enough liquid and remaining in the upright position thereafter at least 15 minutes. In our case, the probable cause of pill esophagitis is taking the pill with little or no fluid prior to bedtime followed by recumbency. Therefore, if you take the pill with inadequate water just before going to bed every drug may cause pill-induced esophagitis.

The best treatment for pill esophagitis is prevention. Patients should be instructed to drink at least 100 - 150 ml. of liquid with any pill. All medications should be taken when the patient is in an upright position, and the patient should remain upright for several minutes after medication ingestion.
